# Supplementary material for: A novel method for reliably measuring miniature and spontaneous postsynaptic events in whole-cell patch clamp recordings in the central nervous system
Source: Front Cell Neurosci. 2025 Jun 18;19:1598016. doi: 10.3389/fncel.2025.1598016 (PMC12213822; doi:10.3389/fncel.2025.1598016)
Supplement: Supplementary file 1 [file Data_Sheet_1.docx]

Supplementary Material

# Supplementary Results

## Research literature measuring minis in the central nervous system: Extended report

There were 107 studies in total that matched our criteria. All of these studies used voltage clamp as the electrophysiological method for recording spontaneous and miniature postsynaptic events. We surveyed the software they used to detect these events. The biggest group, ~48%, reported using MiniAnalysis. 26% reported using Clampfit, while 13% used a custom algorithm. The remaining 13% did not specify. We compared the performance of the two most popular detection algorithms, MiniAnalysis and Clampfit, both commercially available, with our new algorithm ‘minis’.

35 studies (ca. 32%) reported using a current amplitude detection threshold of which 19 studies specified actual numeric value used (-6.8 ± s.d. 0.44 pA, range of -3 to -20 pA). It is likely that a higher fraction of studies made use of amplitude thresholds as only two explicitly reported not using any detection thresholds. Most of the studies did not give enough details (65% of all studies) to judge one way or the other. However, studies that used MiniAnalysis for detection must have used a detection threshold, as this software requires it.

Out of all the studies, 18 carried out research in neocortical pyramidal cells looking at mPSCs only (spontaneous PSCs excluded). 12 studies reported a mean mEPSC amplitude value of -11.74 ± s.d. 0.77 pA (range of -6.0 to -20.9 pA). 13 studies reported a mean incidence rate value of 3.6 ± s.d. 0.34 per second (range of 1.0 to 8.0 per second).

The total putative excitatory synapse count on a single cortical pyramidal cell can number in the tens of thousands (Eyal et al., 2018b). Layer 2/3 pyramidal cells in rats are thought to have 5,000 to 30,000 putative excitatory synapses (Larkman, 1991; Eyal et al., 2018b). Large-thick-trunk/tufted layer 5 pyramidal cells would have an even larger number of contacts (Larkman, 1991). Therefore, we can roughly assume that for most cortical pyramidal cells the number of putative excitatory synapses ranges between 5,000 and 50,000. Conservatively, we could assume that only a minority, roughly 40%, of them are functional (‘non-silent’) synapses with active zones and post-synaptic densities containing AMPA receptors (Motta *et al.*, 2019; Holler *et al.*, 2021). Given that a spontaneous vesicle release at a single release site has been estimated to occur with an approximate incidence rate of 0.0021 per second (Murthy and Stevens, 1999) and given that there may be at least 2.7 release sites per cortical synapse (Holler *et al.*, 2021), the lower bound incidence rate estimate of such events within a single cortical pyramidal neuron might be expected to range somewhere between ca. 10 minis/s and 100 minis/s (5,000*0.4*0.0021*2.7– 50,000*0.4*0.0021*2.7). Incidence rates values reported in the recent literature are below this predicted range by an order of magnitude, suggesting a significant fraction of smaller minis may commonly be ‘missed’ under some recording conditions. This problem has been observed directly (Nevian et al., 2007), using paired dendritic and somatic whole-cell patch recordings, albeit using voltage recording rather than voltage clamp, recording from large layer 5 neocortical pyramidal neurons.

## Detection under realistic incidence rate conditions (20, 40, 80 minis/s)

Data points used to construct the full v-ROC curve include incidence rate and noise conditions that were not necessarily realistic. Based on our calculations, the range of mEPSP incidence rates one might commonly expect during real recordings from neocortical pyramidal neurons is likely to be between 10 and 100 minis/s (see above and (Dervinis and Major, 2024)); In Figures 5A and B, 20, 40 and 80 minis/s (from right to left) are highlighted by open circles. Just as across the entire ROC curve, the performance of ‘minis’ was superior in this incidence rate range compared to the other two algorithms with MiniAnalysis coming second, and Clampfit being worst, by a substantial margin.

Other measures like true positive and false positive rates as functions of the time to the nearest neighbour and V_m_ rate of change during the rise or decay phases pointed to a very similar conclusion made regarding the full ROC curve. First, we found that ‘minis’ performed better than other algorithms at detecting mEPSPs that were within 10 ms of other mEPSPs and that at longer delays MiniAnalysis performed just as well (Supplementary Figure 1). Meanwhile, Clampfit performed consistently worst across the range of inter-mini intervals, whether measured by d’ (Supplementary Figure 1C) or true positive and false positive rates (Supplementary Figures 1A, B, D, and E). When it came to V_m_ rate of change, ‘minis’ consistently showed the best performance at detecting smEPSPs occurring with the background V_m_ rate of change ranging between -40 and 100 µV/s (Supplementary Figures 2 and 3). This is the range where the vast majority of simulated events occurred (Supplementary Figures 2D and 3D). In terms of individual TPR and FPR (Supplementary Figures 2A and B and 3A and B) and cumulative rates (Supplementary Figures 2D and E and 3D and E), the conclusions are much the same as those made regarding the full virtual ROC curve. Namely, that ‘minis’ had the largest TPR and the smallest FPR with an exception being the TPR in relation to rising background (simulated) membrane potential trends (Supplementary Figures 2A and D), with there being a range (45-100 µV/s) where MiniAnalysis had a slightly higher TPR, but not overall performance measured as d’ (Supplementary Figure 2C). Similar to conditions used to construct the full ROC curve, Clampfit showed consistently the worst performance by all measures. The performance of all three algorithms was better with realistic smEPSP incidence rates (Supplementary Figures 2C and 3C) than over the full ‘virtual’ ROC curve (Figures 8C and 9C). In summary, evaluation of detection of mEPSPs simulated at realistic incidence rates supported conclusions made regarding detection performance under a much broader range of simulation incidence rates.

# Supplementary References

Dervinis, M. and Major, G. (2024) ‘Novel quantal analysis method reveals conservation of average excitatory synaptic charge across cortical pyramidal neurons of different sizes’, *bioRxiv* [Preprint]. Available at: https://doi.org/10.1101/2024.07.05.602190.

Eyal, G. *et al.* (2018) ‘Human Cortical Pyramidal Neurons: From Spines to Spikes via Models’, *Frontiers in Cellular Neuroscience*, pp. 1–24.

Holler, S. *et al.* (2021) ‘Structure and function of a neocortical synapse’, *Nature*, 591(7848), pp. 111–116. Available at: https://doi.org/10.1038/s41586-020-03134-2.

Larkman, A.U. (1991) ‘Dendritic morphology of pyramidal neurones of the visual cortex of the rat: III. Spine distributions’, *Journal of Comparative Neurology*, 306(2), pp. 332–343. Available at: https://doi.org/https://doi.org/10.1002/cne.903060209.

Motta, A. *et al.* (2019) ‘Dense connectomic reconstruction in layer 4 of the somatosensory cortex’, *Science*, 366(6469), p. eaay3134. Available at: https://doi.org/10.1126/science.aay3134.

Murthy, V.N. and Stevens, C.F. (1999) ‘Reversal of synaptic vesicle docking at central synapses’, *Nature Neuroscience*, 2(6), pp. 503–507. Available at: https://doi.org/10.1038/9149.

# Supplementary Figure Captions

**Supplementary Figure 1**: Performance when detecting moderately-sized (~0.3 mV) smEPSPs as a function of time to the nearest neighbour with realistic mini rates (20, 40, 80 minis/s). Paler shaded colours indicate 95% confidence intervals.

(A) True positive rate (TPR).

(B) False positive rate (FPR).

(C) Sensitivity index d’ (undefined but arbitrarily high for TPR > 0 and FPR = 0, e.g. for inter-mini intervals below 1 ms, for ‘minis’ algorithm).

(D) Cumulative TPR.

(E) Cumulative FPR.

**Supplementary Figure 2**: Performance when detecting moderately-sized (~0.3 mV) smEPSPs on the rising trend V_m_ phases with realistic minis’ rates (20, 40, 80 minis/s, as in Fig. 9), as a function of the rate of change of V_m_. Pale shaded colours indicate 95% confidence intervals.

(A) True positive rate (TPR)

(B) False positive rate (FPR).

(C) Sensitivity index d’.

(D) Cumulative TPR.

(E) Cumulative FPR.

**Supplementary Figure 3**: Performance when detecting moderately-sized (~0.3 mV) smEPSPs on the membrane potential decay phase with realistic minis rates (20, 40, 80 minis/s, as in Figs. 9 and 10). Pale shaded colours indicate 95% confidence intervals.

(A) True positive rate (TPR) when detecting moderately-sized smEPSPs on the V_m_ decay phase as a function of the V_m_ rate of change.

(B) False positive rate (FPR).

(C) Sensitivity index d’.

(D) Cumulative TPR.

(E) Cumulative FPR.

# Supplementary Tables

|  | Condition | Minis vs. truth | MiniAnalysis vs. truth | Clampfit vs. truth |
| --- | --- | --- | --- | --- |
| Amplitudes | Type 1 All | 1.01×10^-7^ | 3.91×10^-12^ | 7.49×10^-10^ |
|  | Type 1 HI | 3.49×10^-13^ | 5.84×10^-17^ | 1.39×10^-12^ |
|  | Type 1 RI | **0.17** | 8.89×10^-6^ | 0.00021 |
|  | Type 2 | 1.18×10^-5^ | 7.48×10^-9^ | 1.26×10^-7^ |
|  | Real data | - | - | - |
| 10-90% rise times | Type 1 All | 1.33×10^-11^ | 5.03×10^-15^ | 1.54×10^-17^ |
|  | Type 1 HI | 4.68×10^-16^ | 4.33×10^-21^ | 9.95×10^-19^ |
|  | Type 1 RI | 4.14×10^-10^ | 2.16×10^-10^ | 5.47×10^-15^ |
|  | Type 2 | 1.31×10^-13^ | 2.13×10^-10^ | 5.98×10^-20^ |
|  | Real data | - | - | - |
| Decay times | Type 1 All | **0.3** | 0.0006 | 0.0002 |
|  | Type 1 HI | 0.006 | 0.002 | 0.0002 |
|  | Type 1 RI | **0.91** | 1.07×10^-5^ | 0.0002 |
|  | Type 2 | **0.97** | 3.02×10^-6^ | 6.61×10^-5^ |
|  | Real data | - | - | - |
| Incidence rates | Type 1 All | 2.09×10^-5^ | 1.4×10^-18^ | 3.93×10^-27^ |
|  | Type 1 HI | 3.31×10^-13^ | 2.64×10^-30^ | 3.6×10^-37^ |
|  | Type 1 RI | **0.835** | 6.6×10^-5^ | 6.7×10^-15^ |
|  | Type 2 | **0.852** | 1.12×10^-6^ | 4.96×10^-22^ |
|  | Real data | - | - | - |

**Supplementary Table 1:** T-test p-values for mean amplitude, 10-90% rise time, decay time, and incidence rate values of detected minis using different algorithms. Repeated measures t-tests were used when comparing performance of two algorithms and single sample t-tests were used when comparing mean values with ground truth (subtracting ground truth values). Abbreviations are the same as in Table 1.
