# Supplementary figures and images for: A novel method for reliably measuring miniature and spontaneous postsynaptic events in whole-cell patch clamp recordings in the central nervous system
Source: Front Cell Neurosci. 2025 Jun 18;19:1598016. doi: 10.3389/fncel.2025.1598016 (PMC12213822; doi:10.3389/fncel.2025.1598016)

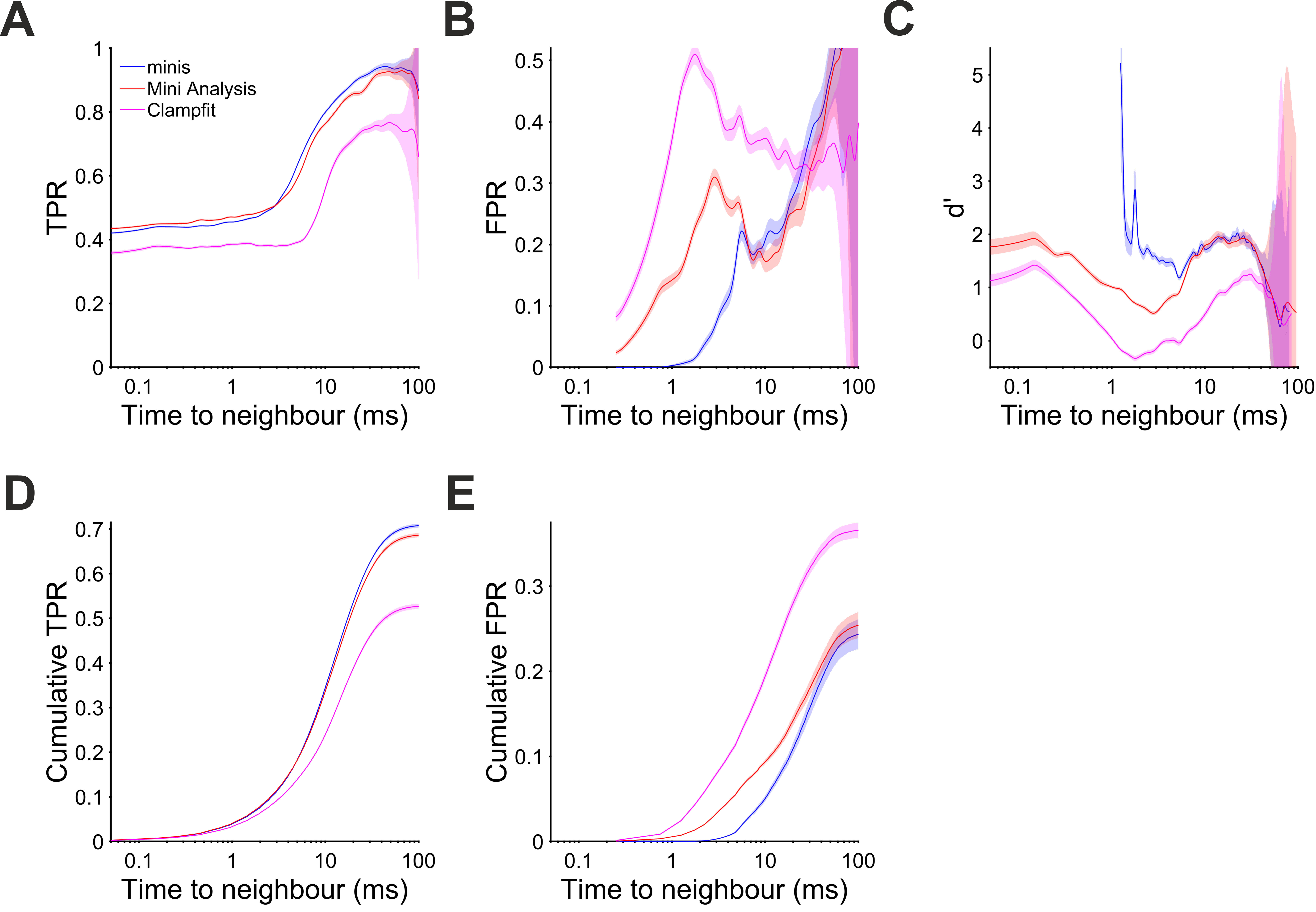

Supplement: Supplementary file 2 [file Image_1.jpg]

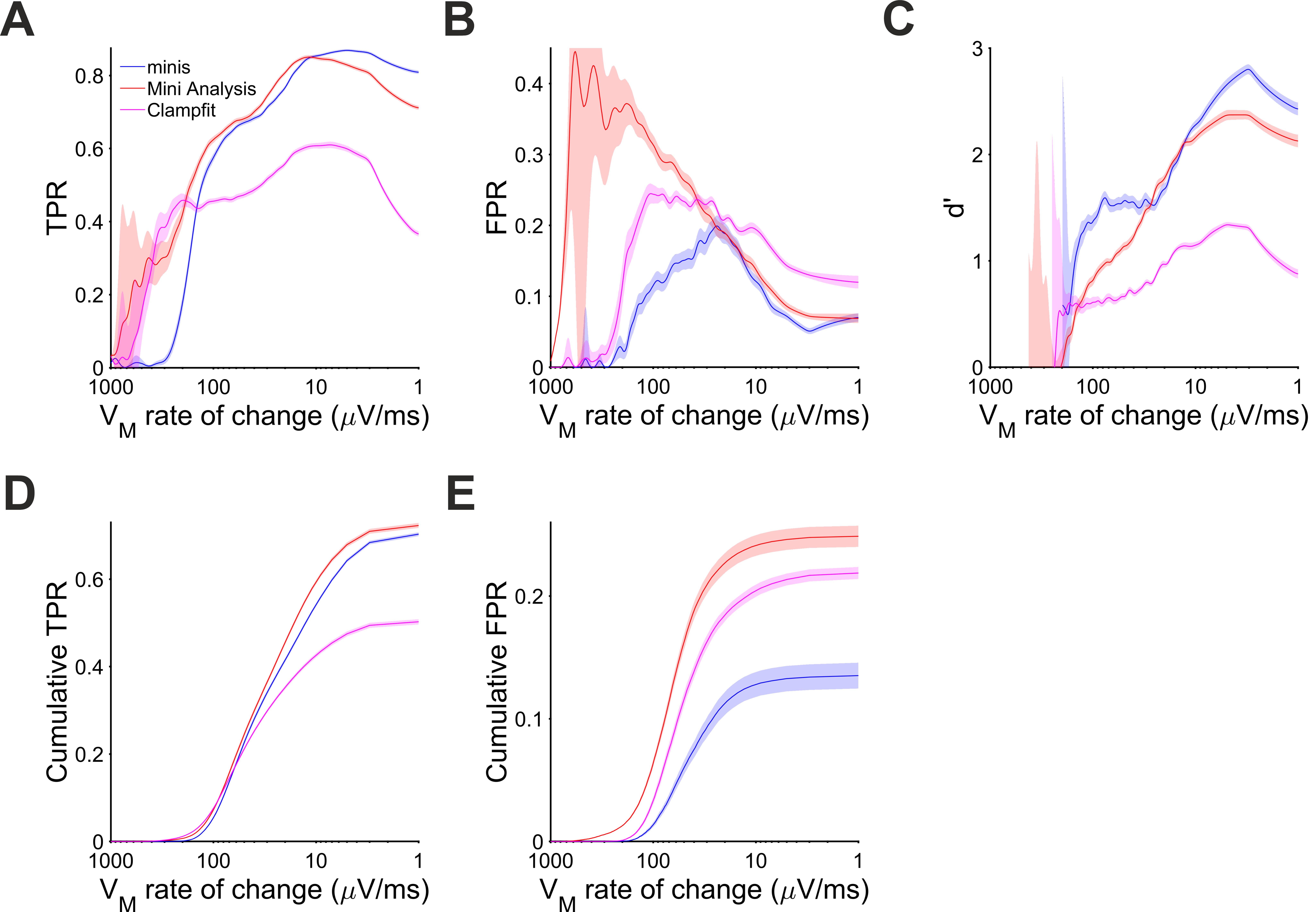

Supplement: Supplementary file 3 [file Image_2.jpg]

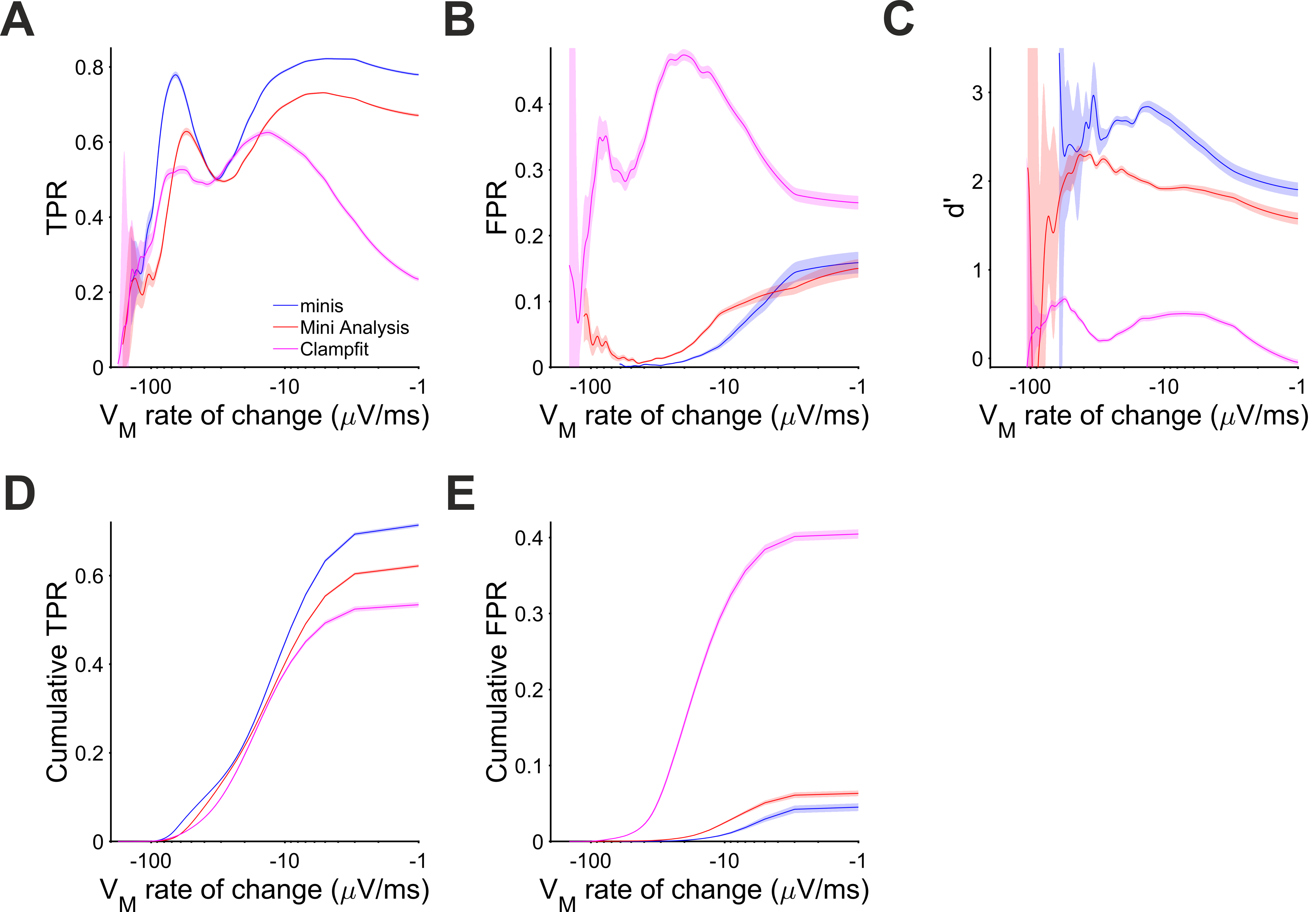

Supplement: Supplementary file 4 [file Image_3.jpg]
